# Use of wearable cardioverter‐defibrillator in association with catheter ablation for atrial fibrillation‐related tachycardiomyopathy

**DOI:** 10.1002/ccr3.2089

**Published:** 2019-04-10

**Authors:** Sergio Conti, Vito Bonomo, Antonio Taormina, Umberto Giordano, Giuseppe Sgarito

**Affiliations:** ^1^ ARNAS Ospedale Civico ‐ Di Cristina ‐ Benfratelli Palermo Italy; ^2^ University of Tor Vergata Rome Italy; ^3^ University of Palermo Palermo Italy; ^4^ University of Messina Messina Italy

**Keywords:** atrial fibrillation, catheter ablation, implantable cardioverter defibrillator, wearable cardioverter defibrillator

## Abstract

Implantable cardioverter‐defibrillator (ICD) is an effective therapy in patients known to be at high risk for sudden cardiac death (SCD). Nevertheless, ICD implantation is not indicated in transient or reversible causes of SCD. Wearable cardioverter‐defibrillator is increasingly used for SCD prevention in patients with a transient risk of ventricular arrhythmia.

## 1 INTRODUCTION

Implantable cardioverter‐defibrillator (ICD) implantation is not indicated in patients with potentially transient or reversible causes of sudden cardiac death (SCD). Wearable cardioverter‐defibrillator (WCD) is increasingly used for SCD prevention in patients who are temporary at high risk of ventricular arrhythmia. Hereby, we describe a case of tachycardiomyopathy successfully managed with ablation and WCD backup.

Implantable cardioverter‐defibrillators are a Class I indication by American College of Cardiology/American Heart Association/Heart Rhythm Society guidelines to prevent SCD in patients with nonischemic dilated cardiomyopathy, New York Heart Association (NYHA) functional class II and III, left ventricular ejection fraction (LVEF) ≤35% and with a life expectancy of >1 year.^1^ In addition, current guidelines recommend deferring implantation of ICDs for 40 days or three months postmyocardial infarction (MI), depending on whether acute revascularization is achieved.^1^ Recently, the benefits of ICD implantation in patients with DCM have been reconsidered after the results of the DANISH trial.^2^ A global assessment, beyond LVEF, may help to improve the appropriateness of ICD indication by identifying those patients who may benefit more from ICD implantation.

Recently, wearable cardioverter‐defibrillators (WCDs) have emerged as a reasonable choice for patients in whom recovery of the LVEF is expected.^3^ A small subset of patients with atrial fibrillation (AF) experience dilated cardiomyopathy (DCM) also called tachycardiomyopathy. Typically, these patients have rapid ventricular rates and persistent AF. It has been shown that patients with tachycardiomyopathy related to AF benefit from ablation with a significant improvement in the LVEF as well as a reduction in the left ventricular end‐diastolic diameter (LVEDD) and left atrial diameter (LAD).^4^ The outcome of patients with tachycardiomyopathy after catheter ablation did not differ from that of patients without structural heart disease.^4^, ^5^ Hereby, we describe a patient with AF and recurrent relapses of heart failure (HF) who underwent successful AF ablation and postprocedural management with the WCD.

## 2 CASE REPORT

A 55‐year‐old man with history of diabetes mellitus, AF, and family history of sudden death was admitted to the emergency department for worsening HF (NYHA class III). The 12‐lead ECG showed AF with a ventricular rate response of 110 bpm. His medications were furosemide, spironolactone, bisoprolol, ramipril, and digoxin. On admission, a transthoracic echocardiogram was performed showing a LVEF of 25% with global hypokinesia. At first, the patient was stabilized with heart rate control and intravenous (iv) furosemide infusion. The coronary angiography revealed normal coronary arteries. Once the transesophageal echocardiography excluded intracardiac thrombi, electrical cardioversion was attempted. However, even if the patient was loaded with amiodarone iv, ECV was unsuccessful. For this reason, the rate control therapy was optimized and the patient was scheduled for a cardiac magnetic resonance (CMR) to better evaluate the underlying substrate. CMR showed global systolic dysfunction resulted in an LVEF of 30% and no late‐gadolinium enhancement (LGE) throughout the left ventricular myocardium. Considering that the patient had an otherwise not explained dilated cardiomyopathy, we considered this clinical scenario compatible with tachycardiomyopathy and a rhythm control strategy was planned. Patient underwent radiofrequency (RF) catheter ablation of AF according to the latest consensus recommendations.^6^

**Figure 1:**
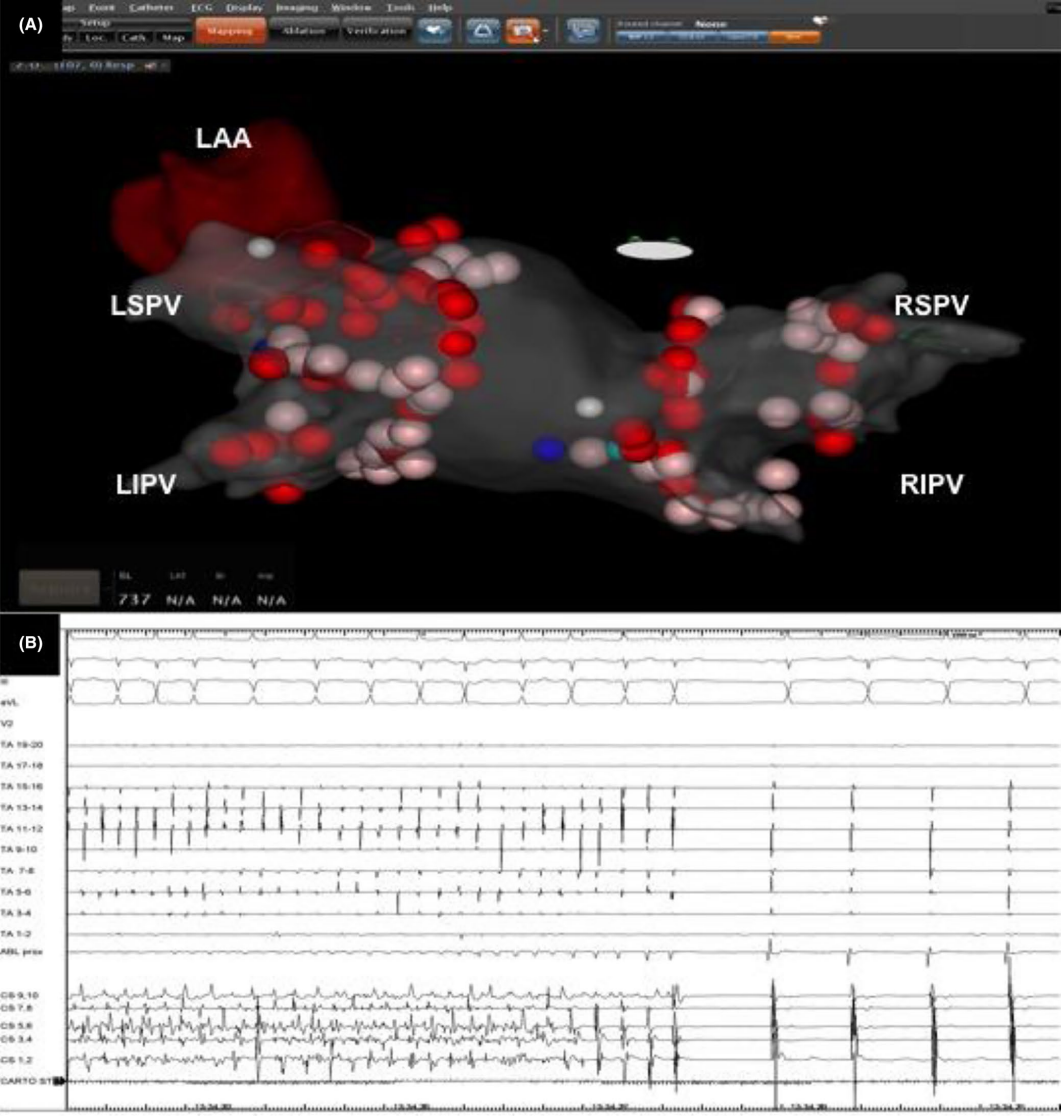
A, Electroanatomical mapping of atrial fibrillation ablation (CARTO). The arrow points to the ridge between the left atrial appendage and the left superior pulmonary vein. LAA, left atrial appendage; LIPV, left inferior pulmonary vein; LSPV, left superior pulmonary vein; RIPV, right inferior pulmonary vein; RSPV, right superior pulmonary vein. B, Atrial fibrillation interruption with sinus rhythm restoration

Briefly, PVI was performed under conscious sedation. A 7F decapolar catheter was inserted into the coronary sinus to guide the transseptal puncture. Transseptal access to the left atrium (LA) was obtained using a Brockenbrough XS needle (Abbott Medical, MN, USA) and an SL1 8.5F transseptal sheath (Abbott Medical, MN, USA). After transseptal puncture, unfractionated heparin was given as bolus (10 000 U) followed by a continuous infusion (1000 U/h) to maintain an ACT ≥350 seconds. The procedure was guided by a 20‐pole circular mapping catheter and electroanatomical mapping system (CARTO 3, Biosense Webster, CA, USA). A 3D shell of the LA and the pulmonary veins (PVs) was created prior to ablation. Pulmonary vein isolation (PVI) was performed using a 3.5 mm bidirectional open irrigated tip catheter with contact force capability (SmartTouch SurroundFlow, Biosense Webster, CA, USA) with an upper temperature limit of 43**°**C, power of 25‐35 W, and an infusion rate of 17 mL/min. A single circumferential lesion was created around the PVs ostia guided by ablation index score. During RF delivery along the ridge between the left superior pulmonary vein and the left atrial appendage, AF interruption with sinus rhythm restoration was documented (Figure 1). Exit and entrance block was sequentially confirmed in each of the PVs using the circular catheter. Far‐field capture and sensing were ruled out using differential pacing maneuvers. Postprocedural echocardiogram showed no pericardial effusion. Two days after the procedure, the patient was fitted with a WCD (LifeVest, Zoll, Pittsburgh, PA, USA), instructed on how to use the WCD by the providing physician and a device representative, and discharged without any complications. At three‐month follow‐up, patient symptoms' significantly improved (NYHA class I), his compliance was satisfactory, and no shocks were recorded. The transthoracic echocardiography showed a complete normalization of the LVEF (55%) and normal end‐diastolic left ventricular volume. At this point in time, the WCD was discontinued and the patient was kept on amiodarone and beta‐blockers and closely followed in the outpatient clinic. At six months, amiodarone was discontinued. The patient had no episodes of atrial and ventricular arrhythmia at 9‐month follow‐up.

## 3 DISCUSSION

A clear causal relationship to explain why some patients with no structural heart disease are more susceptible to develop HF in the setting of a sustained and incessant tachyarrhythmia compared with other patients with chronic arrhythmias has not yet been found. Tachycardiomyopathy has been investigated both in experimental patterns and in humans. Animal models receiving long‐term rapid pacing developed a severe but reversible cardiomyopathy sustained by temporary changes in the function and structure of myocytes.^7^, ^8^ However, it remains unclear whether patients with tachycardiomyopathy have an increased risk for ventricular arrhythmias and sudden cardiac death, and hence deserve an ICD.^9^ A large study including 659 patients undergoing PVI for AF comparing HF patients due to tachycardiomyopathy and controls showed a significantly worse LVEF, larger LAD, and larger LVEDD in patients with tachycardiomyopathy. At 6 months after PVI, the tachycardiomyopathy group patients had a significant improvement of LVEF, LAD, and LVEDD.^4^

The WCD is used to give a transient protection to patients deemed to be at high risk for SCD and to avoid unnecessary ICD implantations.^10^, ^11^ While patients are protected against life‐threatening arrhythmias, pharmacological and nonpharmacological therapies can be optimized. Changes in the clinical status during this “window‐period” allow further risk assessment to decide whether a permanent ICD is needed. Previous data suggest that a significant proportion of patients did not ultimately require ICD implantation suggesting this may be a cost‐effective strategy in patients at risk of SCD.^12^ WCD has been recently evaluated in the multicenter randomized clinical trial Wearable Cardioverter‐Defibrillator after Myocardial Infarction (VEST trial).^13^ This trial aimed to evaluate the potential benefit of WCD in patients who were immediately post‐MI with a depressed LVEF. Since the presentation of the results, the study has been the subject of an intense debate. Over the mean follow‐up, the rate of SCD in the WCD group was 1.6% vs 2.4% in the control group (*P*: 0.18). Although it was not statistically significant, the trend was positive, with a 33% relative reduction in SCD. Moreover, total mortality in the WCD group was 3.1% vs 4.9% in the control group (*P*: 0.04). Thus, there was a 36% relative risk reduction in total mortality that did reach statistical significance. Finally, compliance with WCD in the study group appeared to be lower than observed in clinical practice and in previously reported WCD registry.

The first study that specifically evaluated the effectiveness of the WCD in patients with tachycardiomyopathy related to AF or atrial flutter was recently published.^5^ Although it was a somewhat small study enrolling 130 patients of whom 20 suspected tachycardiomyopathy, the authors found a more favorable outcome in patients with tachycardiomyopathy compared to patients with different indications for WCD therapy and needed less often ICD implantation.

Implantable cardioverter‐defibrillator implantation in patients with DCM has been recently object of the DANISH trial that showed the absence of total mortality reduction in patients randomized to ICD, forcing us to rethink the selection criteria for ICD implantation.^2^ As recently suggested, multiparametric assessment may help to improve the appropriateness of ICD indication by identifying those patients who may benefit more from ICD implantation. Several invasive and noninvasive markers of arrhythmic risk have been proposed. Fibrosis identified by CMR seems to be the most promising marker in DCM patients.^14^ Large prospective studies and meta‐analyses have shown that the absence of fibrosis in DCM patients predicts a relatively low risk of SCD, while the presence of fibrosis predicts a relatively high risk of SCD, irrespective of the EF value.^15^, ^16^, ^17^

Although this clinical scenario was compatible with tachycardiomyopathy, carrying a relatively low risk of SCD, we managed our patient with the WCD. In this case after successful PVI, LVEF significantly improved in sinus rhythm, the patient had no more HF hospitalization, good compliance with the WCD, and furthermore, an unnecessary ICD implantation has been avoided.

## CONFLICT OF INTEREST

None declared.

## AUTHOR CONTRIBUTION

SC: wrote the paper. VB: collected the procedural data. AT: collected the follow‐up data. UG: reviewed the paper. GS: approved the paper.
